# Peroxisomes as redox-signaling nodes in intracellular communication and stress responses

**DOI:** 10.1093/plphys/kiab060

**Published:** 2021-02-15

**Authors:** Luisa M Sandalio, Maria Angeles Peláez-Vico, Eliana Molina-Moya, Maria C Romero-Puertas

**Affiliations:** Department of Biochemistry, Cellular and Molecular Biology of Plants, Estación Experimental del Zaidín-CSIC, Profesor Albareda 1, 18008 Granada, Spain

## Abstract

Peroxisomes are redox nodes playing a diverse range of roles in cell functionality and in the perception of and responses to changes in their environment.

**ADVANCES**

- Peroxisomal H_2_O_2_ regulates pathogen associated processes, DNA repair systems, cell cycles and phytohormone-dependent signalling.
- Peroxisomes regulate cellular processes in the cytosol and other cell compartments through moonlighting proteins such as CAT3, which is able to transnitrosylate and degrade GSNOR via autophagy.
- Peroxisomes are highly dynamic organelles that are capable of changing their number, size, morphology and speed in response to environmental redox changes.
- Peroxules are ROS- and NO-induced dynamic structures that are regulated by PEX11a, which connects peroxisomes to chloroplasts, mitochondria, ER and lipid bodies.
- Under basal and stress conditions, peroxisomal populations and quality are regulated by selective autophagy (pexophagy) which is controlled by ROS and the peroxisomal protease LON2.

**Abstract**

Redox compartmentalization in organelles is an effective evolutionary strategy (Box 1; Jones and Go, 2010). From an evolutionary perspective, peroxisomes, originating from the endoplasmic reticulum (ER), were selected to house a range of metabolic pathways involving the production of certain reactive oxygen species (ROS) such as H_2_O_2_ to avoid toxicity to other organelles such as mitochondria (Gabaldón, 2018). Peroxisomes play a diverse range of roles in cell functionality and in the perception of and responses to changes in their environment (Sandalio and Romero-Puertas, 2015; Lismont et al., 2019). The range of functions associated with plant peroxisomes has increased considerably over the last two decades (Table 1). As most of these pathways produce ROS and nitric oxide (NO), disturbances in these metabolic processes trigger transitory changes in ROS/reactive nitrogen species (RNS) production. These changes regulate peroxisomal metabolism, leading to peroxisome-dependent signaling and organelle crosstalk, which triggers specific cell responses (Sandalio and Romero-Puertas, 2015). The biosynthesis of phytohormones jasmonic acid (JA), auxin IAA, and salicylic acid (SA) associated with the β-oxidation pathway contributes to the complex role of peroxisomes in development and stress responses (Kao et al., 2018; Figure 2A). Peroxisomes dynamically regulate their number, shape, and protein content in response to changing environmental conditions and remain in close contact with other subcellular compartments such as mitochondria and chloroplasts (Sandalio and Romero-Puertas, 2015; Shai et al., 2016; Sandalio et al., 2020). Peroxisomes play a key role in the evolution of the metabolic networks of photosynthetic organisms by connecting oxidative and biosynthetic pathways operating in different compartments. This review updates our knowledge of peroxisomal redox homeostasis and the role of ROS and NO in the functionality, biogenesis and abundance of these organelles, as well as their role as redox hubs in metabolic regulation, signaling, and organelle crosstalk.

**Table 1:** Plant peroxisome functions

ROS and RNS metabolism
H_2_O_2_ and NO signaling
Photorespiration
Phytohormones biosynthesis (JA, IAA, SA)
Fatty acid β-oxidation
Glyoxylate cycle
Polyamine catabolism
Amino acids metabolism
Indole glucosinolates metabolism
Ureide metabolism
Purine catabolism
Biotin biosynthesis
Ubiquinone biosynthesis
Phylloquinone biosynthesis
Isoprenoids biosynthesis
BA derivate biosynthesis
Sulfite metabolism

Box 1 Subcellular redox compartmentalization

As oxygen-dependent redox reactions came to control life after O_2_ appeared in the atmosphere, cells developed complex mechanisms to detect and regulate these changes to maintain metabolic functionality. Redox compartmentalization in organelles is an effective evolutionary strategy, which regulates physiological and stress conditions through site-specific footprinting (Jones and Go, 2010). This redox circuit flexibility facilitates rapid responses to changes in intracellular redox equilibrium, which, in turn, favors beneficial signaling and detrimental oxidative stress. Photosynthetic organisms have developed efficient redox control systems using redox signals as the most fundamental forms of information (Foyer and Noctor, 2016). The thiol/disulphide couples GSH/GSSG and Cys/CySS, the ASC/DHA couple and a broad range of redox dependent proteins, which are counterparts of ROS such as H_2_O_2_ and other oxidants, form the core of the redox state and regulate the cell signaling, structure and activity of proteins and transcription factors. Apart from ROS, RNS are also redox signaling molecules, which include NO and peroxynitrite (ONOO^−^). Both ROS and RNS regulate covalent, often reversible, modifications, mainly targeting Cys, which regulates metabolic shifts and triggers signaling cell responses (Noctor et al., 2018; Sánchez-Vicente et al., 2019). Although irreversible oxidation products, such as sulfonic acid, carbonylation, and nitration, adversely affect proteins and lipids, they may be also involved in oxidative signaling (Foyer et al., 2017). Analyses of redox potential in plant tissue identified peroxisomes as some of the most oxidized cellular organelles, with a redox potential of approximately −360 mV (Bratt et al., 2016; Smirnoff and Arnaud, 2019).

## Peroxisomes are ROS and NO producers

### Peroxisomes produce and scavenge ROS

**Figure 1:**
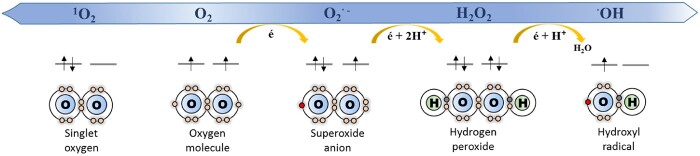
Sequential reduction of O_2_ and ROS production: superoxide (O_2_^.−^), hydrogen peroxide (H_2_O_2_), and hydroxyl (^·^OH) radicals.

**Figure 2:**
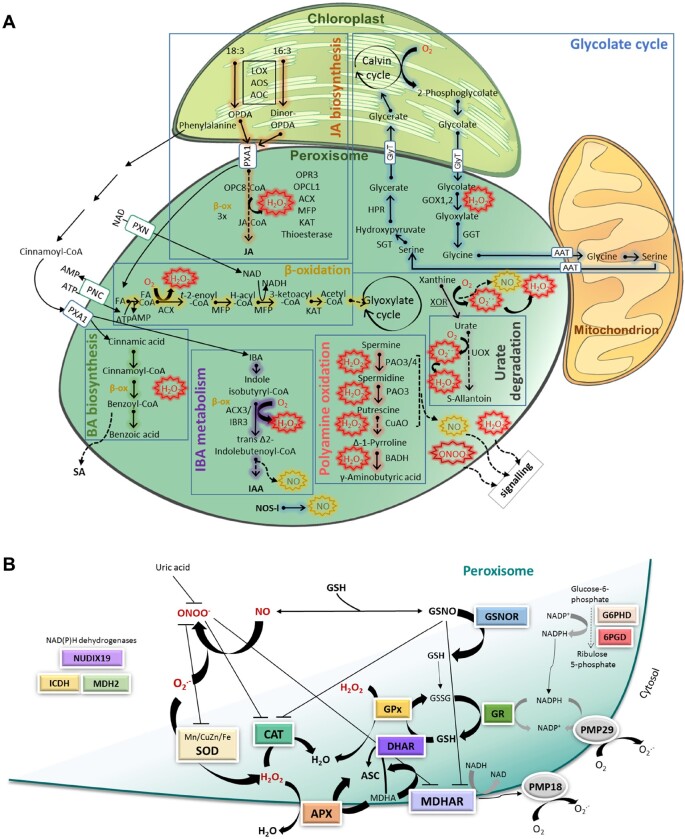
Oxygen and nitrogen reactive species metabolism in peroxisomes. A, Principal peroxisomal metabolic pathways associated with peroxisomal ROS and NO production. ROS are produced in metabolic pathways such as β-oxidation, photorespiration, ureides metabolism, and polyamine oxidation, and in a small electron transport chain associated with the membrane (peroxisomal membrane proteins, PMP18 and PMP29; Figure 2B). NO is produced in peroxisomes by NOS-like (NOS-l) activity, although other sources, such as XOR, polyamine oxidation, and IBA metabolism, could also be involved. ROS, NO, and other RNS may leak out of the peroxisome (dashed arrows) and act as signal molecules that regulate cell metabolism and gene expression. B, Scheme of peroxisomal antioxidant defenses, RNS scavengers, and NAD(P)H supply. O_2_^.−^ is regulated by SODs, while H_2_O_2_ is controlled by CAT, the ASC-GSH cycle, and GPx. Peroxynitrite (ONOO^−^) and GSNO are produced in peroxisomes by reaction of NO with O_2_^.−^ and glutathione (GSH), respectively. GSNO can negatively regulate MDHAR and CAT through *S*-nitrosylation and nitration, and SOD may be regulated by nitration. SOD may indirectly control ONOO^−^ by regulating O_2_^.−^ levels. Uric acid acts as an ONOO^−^ scavenger. NAD(P)H is supplied by the oxidative pentose phosphate pathway (G6PD; 6PGD), ICDH, MDH, and NUDIX19. 6PGD, 6 phosphogluconate dehydrogenase; AAT, amino acid translocator; AOC, allene oxide cyclase; AOS, allene oxide synthase; APX, ascorbate peroxidase; BADH, betaine aldehyde dehydrogenase; CAT, catalase; CuAO, copper amine oxidase1; DHAR, dehydroascorbate peroxidase; GOX1,2, glycolate oxidase1,2; G6PD, glucose-6-phosphate dehydrogenase; GGT, glutamate–glyoxylate aminotransferase; GlyT, glycerate–glycolate translocator; GR, glutathione reductase; GPx, glutathione peroxidase; H-Acyl-CoA, 3-hydroxyacyl-CoA; HPR, hydroxypyruvate reductase; IAA, indole-3-acetic acid; IBA, indole-3-butyric acid; IBR3, acyl-coA dehydrogenase/oxidase-like IBR3; ICDH, isocitrate dehydrogenase; KAT, L-3-ketoacyl-CoA-thiolase; LOX, lipoxygenase; MDH2, malate dehydrogenase; MDHAR, monodehydroascorbate peroxidase; MFP, multifunctional protein; OPCL1, OPC-8:0 CoA ligase1; NOS-l, NO synthase-like; NUDIX19, nudix hydrolase homolog 19; OPR3, OPDA reductase3; PAO3, polyamine oxidase3; PAO3/4, polyamine oxidase 3/4; PNC, peroxisomal ATP carrier; PXA1, peroxisomal ABC-transporter1; PXN, peroxisomal NAD carrier; SGT, serin–glyoxylate aminotransferase; UOX, urate oxidase.

ROS include an array of molecular oxygen derivatives that occur as a normal attribute of aerobic life (Figure 1). Peroxisomes are one of the main sources of cellular ROS production and one of the most oxidized cellular organelles (Smirnoff and Arnaud, 2019). However, peroxisomes have a complex antioxidant system to balance ROS levels, enabling them to strictly regulate organelle functionality, metabolism, and signaling networks. The first step in O_2_ univale O_2_^.−^ (Figure 1), is produced in ureide and nucleic acid catabolism by xanthine oxidoreductase (XOR) and urate oxidase or uricase (UO) reaction (Werner and Witte, 2011; Sandalio et al., 2013; Figure 2A); in the sulfite oxidation by sulfite oxidase (SO; Byrne et al., 2009); and in the NADH/NADPH-dependent electron transport chain in the peroxisomal membrane. Superoxide accumulation is regulated by different superoxide dismutases (SOD; reviewed in Sandalio and Romero-Puertas, 2015; Figure 2B).

In photosynthetic tissue, peroxisomes accumulate the highest concentrations of organelle H_2_O_2_ (the second step in O_2_ reduction) with a flux of ∼10,000 nmol^−2^ m^−2^ s^−1^ (Foyer and Noctor, 2003). The use of H_2_O_2_ ratiometric reporter HyperAs targeting peroxisomes has facilitated the imaging of changes in peroxisomal H_2_O_2_ accumulation in response to Cd treatment (Calero-Muñoz et al., 2019) and the increase in intraperoxisomal Ca^2+^ levels (Costa et al., 2010). The main source of H_2_O_2_ in peroxisomes in green tissue is GOX in the photorespiration cycle (Figure 2A), which contributes up to 70% of total H_2_O_2_ production in plant cells (Reumann and Weber, 2006; Foyer et al., 2009). Photorespiration requires coordination of the chloroplast, peroxisome, mitochondrion, and cytosol; and photorespiration-dependent H_2_O_2_ production increases considerably under environmental stress conditions such as heat and drought (Talbi et al., 2015; Walker et al., 2016), heavy metal (Gupta et al., 2017), high light (Cui et al., 2016a), and biotic stress (Rojas et al., 2012; Hodges et al., 2016; Yang et al., 2018). Fatty acid β-oxidation, another source of H_2_O_2_ in peroxisomes by the Acyl-CoA oxidase (ACX), provides energy during the initial stage of seedling growth by oxidizing fats stored as triacylglycerol (TAG) in oil bodies (Rinaldi et al, 2016; Figure 2A). Other β-oxidation pathways are active in green tissues, including the synthesis of ubiquinone, hormones such as indole acetic acid (IAA) and JA, and secondary metabolites such as benzoic acid (BA) and phenylpropanoids (reviewed in Pan et al., 2020; Figure 2A). Polyamine catabolism and sarcosine oxidase are additional peroxisomal sources of H_2_O_2_ (Figure 2A; Goyer et al., 2004; Wang et al., 2019). Peroxisomal H_2_O_2_ levels are regulated by balancing H_2_O_2_ generation and scavenging rates (Figure 2B) by catalase (CAT), which account for 10%–25% of total peroxisomal proteins (Reumann et al., 2004; Figure 2B). Arabidopsis (*Arabidopsis thaliana*) plants contain three *CAT* genes, *CAT1*, *CAT2*, and *CAT3*, with CAT2 being the most important defense against photorespiratory H_2_O_2,_ accounting for 80% of activity (reviewed in Mhamdi et al., 2012). In fact, physical GOX-CAT interactions regulated by SA occur in rice leaves (Zhang et al., 2016). A protective association between CAT and isocitrate lyase has also been observed in castor bean glyoxysomes (Yanik and Donaldson, 2005) and CAT2 also interacts with ACX2/ACX3 regulating their activity and therefore the SA-mediated regulation of JA biosynthesis, under biotrophic infection (Yuan et al., 2017). Although the extraordinarily low affinity of CAT for H_2_O_2_, with a Km of around 43 mM, reduces its efficiency in controlling H_2_O_2_ the abundance of CAT compensates for this low affinity (Foyer and Noctor, 2016). The peroxisomal ascorbate–glutathione cycle, which, in Arabidopsis, composed of ascorbate peroxidase (*APX3* and *APX5*), monodehydroascorbate reductase (*MDHAR1*), dehydroascorbate reductase (*DHAR1*), and glutathione reductase (*GR1*; reviewed in Mhamdi et al., 2012; Sandalio and Romero-Puertas 2015; Pan et al., 2020; Figure 2B) also contribute to H_2_O_2_ homeostasis. MDHAR and APX are associated with the peroxisomal membrane and the higher afﬁnity for H_2_O_2_ of APX (100 μM) as compared to CAT, could regulate H_2_O_2_ leakage from peroxisomes to the cytosol (Del Río et al., 2003; Kaur et al., 2009; Eastmond, 2007; Figure 2B). Therefore, CAT and APX are positioned to enable H_2_O_2_ to act as a second messenger. Glutathione S-transferases support peroxide regulation in these organelles (Pan and Hu, 2018). The ascorbate–glutathione cycle also facilitates regeneration and maintenance of the peroxisomal redox buffers ASC/DHA and GSH/GSSG. The use of ratiometric glutathione redox potential reporters, such as roGFP2, targeting peroxisomes has facilitated the imaging of peroxisome oxidation under extended dark stress and the application of elicitors (Bratt et al., 2016).

### Peroxisomal NO/RNS production and scavenging

Although NO is a well-known signaling molecule in plants, its metabolism has not been fully elucidated (León and Broseta, 2020). Peroxisomal NO production has been associated with a NO synthase-like activity (NOS-l; Figure 2A; Barroso et al., 1999), the conversion of IBA to IAA by β-oxidation (Figure 2A;Schlicht et al., 2013), polyamine catabolism (Figure 2A; Wimalasekera et al., 2011; Agurla et al., 2018), and the XOR reaction (Figure 2A; Antonenkov et al., 2010; Wang et al., 2010). Other nitrogen-derived species, such as peroxynitrite (ONOO^–^), resulting from the $O_{2}^{-}$/NO reaction, and nitrosoglutathione (GSNO), resulting from the combination of NO and GSH and considered a cellular NO reservoir, have been detected in peroxisomes (Figure 2A; Ortega-Galisteo et al., 2012; Corpas and Barroso, 2014). Peroxisomal SOD could regulate ONOO^–^ accumulation by controlling O_2_^.−^ availability and CAT could degrade it, as reported in animal cells (Gebicka and Didik, 2009), and thus play a key modulatory role at the cross-point between H_2_O_2_ and NO/ONOO^–^-mediated signaling pathways (Figure 2B). Urate, a well-known peroxynitrite scavenger (Hooper et al., 2000; Alamillo and García-Olmedo, 2001), may contribute also to regulate ONOO^–^ in peroxisomes (Figure 2B). *S*-nitrosoglutathione reductase (GSNOR), which balances NO and *S*-nitrosothiol levels, has been proteomically identified in plant peroxisomes (reviewed in Sandalio et al., 2019), although this requires validation.

### NADH/NADPH regeneration in peroxisomes

The concept of redox stress (oxidative and reductive) reflected by changes in NAD(H)/NADP(H) has gained increasing attention. The NAD(P)H cofactor is required to β-oxidation and antioxidative defenses MDHAR and GR. NAD(P)H regeneration in peroxisome take place by the oxidative pentose phosphate pathway (OPPP; Corpas et al., 1999; Reumann et al., 2007; Lansing and Doering, 2020; Figure 2B), NADP-dependent isocitrate dehydrogenase (ICDH; Corpas et al., 1999; Reumann et al., 2007; Figure 2B), NADH phosphorylation by NADH kinase 3 (NADK3; Waller et al., 2010) and possibly betaine aldehyde dehydrogenase (ALDH19; Hou and Bartels, 2015). The peroxisomal NADH pool is supported by malate dehydrogenase MDH2 (Cousins et al., 2008; Figure 2B). Peroxisomes also contain pyrophosphatase Nudix Hydrolase Homolog 19 (NUDT19), which hydrolyzes NADPH to NMNH, as well as 2′,5′-ADP and NADH to NMNH and AMP (Lingner et al., 2011).

## ROS- and NO-dependent PTMs in peroxisomal metabolism regulation

Analysis of peroxisomal proteomes shows that a large number of peroxisomal proteins (35%) are targeted by multiple PTMs (Sandalio et al., 2019). Peroxisomal-dependent ROS/RNS can fine-tune post-translational redox changes in proteins, regulating stability, activity, location, and protein–protein interactions (Duan and Walther, 2015; Hashiguchi and Komatsu, 2016; Sandalio et al., 2019; Foyer et al., 2020) supporting peroxisomes capacity to regulate their metabolism and dynamics in response to environmental changes. Hydrogen peroxide leads to rapid and reversible oxidative protein modifications such as sulfenylation, sulfinylation, and intra- and intermolecular disulfide bond formation, which contribute to coordinated regulation of cellular processes, while overoxidation by sulfonylation appears to be an irreversible process (reviewed in Noctor et al., 2018; Young et al., 2019; Sandalio et al., 2019; Sies and Jones, 2020). Given their transient nature, these sulfur modifications are regarded as redox switches (Huang et al, 2018). Peroxisomal antioxidant defenses, fatty acid β-oxidation, and photorespiration are prone to H_2_O_2_-dependent redox regulation (reviewed in Sandalio et al., 2019). The glyoxalase 1 (GLX1) homolog is a putative sulfenylated protein involved in protection against carbonyls (Schmitz et al., 2018).

NO, in turn, modifies proteins through covalent PTMs including *S*-nitrosylation (Martínez-Ruiz et al., 2011; Sánchez-Vicente et al., 2019). Putative peroxisomal *S*-nitrosylated proteins also include antioxidants and enzymes from the photorespiration cycle (Romero-Puertas and Sandalio, 2016; Sandalio et al., 2019) suggesting that *S*-nitrosylation plays an important role in regulating peroxisomal H_2_O_2_ concentrations under physiological and stress conditions (Ortega-Galisteo et al., 2012). Recently, the noncanonical catalase CAT3, identified as a “repressor of” GSNOR1 (ROG1), was reported to transnitrosylate GSNOR1 to promote its degradation by autophagy, while CAT1 and CAT2 do not do it, thereby CAT3 positively regulates NO signaling and according to Arabidopsis *rog1* mutants are more susceptible to NO than WT (Chen et al., 2020). CAT3 is localized in peroxisomes, the cytoplasm, and the plasma membrane (Li et al., 2015; Zou et al., 2015) and is recruited into the nucleus by the cucumber mosaic virus (CMV) 2b protein (Inaba et al., 2011; Murota et al., 2017). Zhan et al. (2018) have reported that *S*-nitrosylation induces selective autophagy of Arabidopsis GSNOR1 during hypoxia responses. CAT3 also interacts with other proteins in the cytosol and plasma membrane, thus increasing the likelihood that these proteins are also substrates of CAT3 transnitrosylase activity (Chen et al., 2020). These findings suggest NO self-regulation and ROS/NO crosstalk. Zhang et al. (2020) have reported that glutathione denitrosylation is required to maintain the upregulation of GSNOR activity; thus coordinating GSNOR activity with protein *S*-nitrosylation levels to ensure appropriate signaling involving the SA pathway in response to H_2_O_2_.

Some fatty acid β-oxidation enzymes, including ACX2, 3, may be *S*-nitrosylation targets (Sandalio et al., 2019). OPC-8:0 CoA Ligase1 (OPCL1), involved in activating JA biosynthetic precursors in leaf peroxisomes (Koo et al., 2006), is also a putative target of *S*-nitrosylation, pointing to NO/JA-crosstalk. Proteomic analyses suggest that BRI1 suppressor 1 (BSU1)-like 3 is targeted by *S*-nitrosylation (Sandalio et al., 2019) suggesting NO-dependent brassinosteroids signaling. NO-dependent nitration also inhibits peroxisomal antioxidants such CAT (Lozano-Juste et al., 2011; Chaki et al., 2015) and SOD (Holzmeister et al., 2015). Therefore, NO and ROS, apart from self-regulation (Romero-Puertas and Sandalio, 2016), may also regulate specific proteins and/or metabolic pathways and metabolite channeling, depending on the redox environment both inside and outside the peroxisome.

## Peroxisome-dependent redox regulation of transcriptional responses

ROS act as secondary messengers that are sensed by specific redox-sensitive proteins, which activate signal transduction pathways and alter gene expression (Suzuki et al., 2012; Mittler, 2017). Different ROS trigger different protein modifications, as shown by different gene expression patterns (Møller and Sweetlove, 2010; Mor et al., 2014). The subcellular site, where the ROS/oxidation state is modified, acts as a specific cellular redox network signal (Foyer and Noctor, 2003; König et al., 2012; Foyer et al., 2017) and leaves a specific imprint on the transcriptome response (Rosenwasser et al., 2011). The selective reactivity, stability, and diffusibility of H_2_O_2_ make it an ideal signaling molecule (Sewelam et al., 2014; Sies and Jones, 2020). Mutants lacking peroxisomal CAT2 (*cat2*) have been extensively studied in Arabidopsis and tobacco (*Nicotiana tabacum*) plants under control and stress conditions, showing that altering peroxisomal H_2_O_2_ induces changes in gene expression profiles (Vandenabeele et al., 2003, 2004; Chaouch et al., 2010; Queval et al., 2012). This profile showed specificity with transcriptional responses that differ from those induced by chloroplast-derived H_2_O_2_ (Sewelam et al., 2014). Analyses of WT plants grown at specific atmospheric CO_2_ levels to boost photorespiration and production of H_2_O_2_ (Chaouch et al., 2010; Queval et al., 2012) and of WT plants treated with aminotriazole, a catalase inhibitor (Gechev et al., 2005), have also shown that peroxisomal H_2_O_2_ plays a role in signaling, as a transcriptomic footprint have been linked to peroxisomes (Rosenwasser et al., 2013). However, little is known about how peroxisome-derived H_2_O_2_ coordinates or relays signaling events. Although peroxisome-dependent gene regulation involves several metabolic categories (reviewed in Sandalio and Romero-Puertas, 2015; Su et al., 2019), those related to protein repair responses under stress conditions are regulated in *cat2* mutants (Queval et al., 2007; Sewelam et al., 2014); suggesting that peroxisomes are involved in acclimation and survival processes under changing environmental conditions. The triple mutant *cat1 cat2 cat3* shows serious redox disturbance and growth defects under physiological conditions, with differentially expressed genes belonging to plant growth regulation, as well as abiotic and biotic stress response categories. Some of these genes belong to transcription factor and receptor-like protein kinase categories (Su et al., 2019). The ROS signals derived from different cell compartments are proposed to connect in the cytoplasm with MAPK pathway to regulate the expression of nuclear genes (Noctor and Foyer, 2016). Several genes related to MAPK cascade pathways, such as MPK11, MPK13, and serine/threonine kinase oxidative signal inducible 1 (OXI1), are severely altered in the triple *cat* mutant (Su et al., 2019). Thus, peroxisomal H_2_O_2_ appears to participate in retrograde signaling, although little is known about the underlying molecular mechanisms and crosstalk with ROS from other compartments (Sandalio and Romero-Puertas, 2015; Su et al., 2019).

## Redox-dependent regulation of peroxisomal plasticity

### Peroxisome biogenesis and proliferation

**Figure 3:**
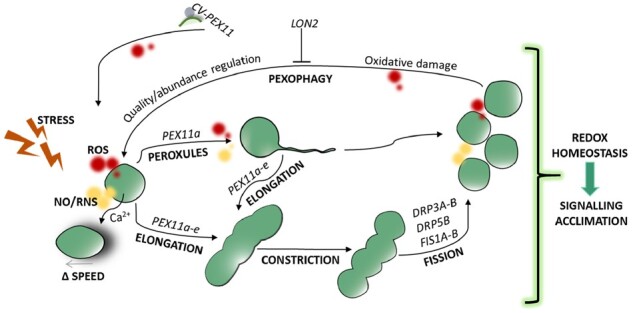
Hypothetical scheme showing changes in peroxisomal dynamics and their regulation, as well as their contribution to cell responses to abiotic stresses such as metal toxicity. Cd stress promotes the generation of ROS and NO, which activate peroxins (PEX11a and PEX11e), probably through ROS-/NO-dependent post-translational modifications (PTMs). PEX11a promotes the formation of peroxules, which may control ROS/NO accumulation and ROS-dependent gene expression. Peroxisomal elongation, constriction, and proliferation, which are regulated by ROS and NO, were later observed. Longer exposure periods increase the speed of peroxisome movement (Δ SPEED), which is also controlled by ROS. The number of peroxisomes, as well as oxidized, damaged peroxisomes, can be regulated by pexophagy or via a process independent of autophagy involving chloroplast vesicle interactions with PEX11 (CV-PEX11), both of which processes are regulated by ROS. All these changes in peroxisomal dynamics may be involved in redox homeostasis and redox-dependent signaling, leading to plant acclimation to the stress. Red color, ROS; yellow color, NO. DRPs, dynamin-related proteins; FIS1A-B, fission protein1A-B; LON2, lon protease homolog 2.

Their high plasticity enables peroxisomes to adapt their number, morphology, movement, and metabolic pathways to changes in their environment (Figure 3). However, why certain signals and molecules trigger these changes, when these changes occur, and how dynamic peroxisomal changes function in relation to tolerance are not well understood. Some evidence shows that peroxisomal proliferation through the division of preexisting peroxisomes is regulated by ROS (López-Huertas et al., 2000; Figure 3; Box 2). Peroxisome proliferation occurs in response to abiotic stresses associated with ROS production: ozone (Oksanen et al., 2004), clofibrate (Nila et al., 2006; Castillo et al., 2008), salinity (Mitsuya et al., 2010; Fahy et al., 2017), cadmium (Romero-Puertas et al., 1999; Rodríguez-Serrano et al., 2016), drought (Ebeed et al., 2018), ABA (Ebeed et al., 2018), and senescence (Pastori and del Río, 1997). Interestingly, NO has recently been reported to be involved in regulating peroxisome proliferation in response to Cd (Terrón-Camero et al., 2020). Proteins involved in peroxisome biogenesis and maintenance are called peroxins (PEXs; Baker et al., 2016; Reumann and Bartel, 2016; Kao et al., 2018; Reumann and Chowdhary, 2018). Peroxisome proliferation under abiotic stress appears to be regulated by specific PEX11 genes which contain five members (*PEX11a-PEX11e*; Lingard and Trelease, 2006; Orth et al., 2007) and under abiotic stressspecific *PEX11* appears to regulate peroxisome proliferation. Thus, salinity upregulated *PEX11a* and *PEX11c* in *A. thaliana* (Fahy et al., 2017), while *PEX11a* and *PEX11e* were upregulated in response to Cd exposure in Arabidopsis plants (Rodríguez-Serrano et al., 2016; Terrón-Camero et al., 2020), and *PEX11b*, *PEX11c*, and *PEX11d* were upregulated by hypoxia (Li and Hu, 2015). Gene coexpression analysis in Arabidopsis plants under drought conditions shows clustering of photorespiratory genes and peroxisomal abundance, suggesting that H_2_O_2_ plays a role in peroxisomal abundance regulation (Li and Hu, 2015). This is supported by the absence of peroxisome proliferation in *gox2* Arabidopsis mutants exposed to Cd (Calero-Muñoz et al., 2019). However, genome analyses of *Physcomitrium*, *A. thaliana*, and *Triticum aestivum* show upregulation of β-oxidation in response to drought, dehydration, and ABA (Ebeed et al., 2018). Interestingly, *PEX11* gene family expression differs between drought-sensitive and resistant wheat genotypes, although the significance or not of these differences for drought tolerance has not been established (Ebeed et al., 2018). These findings suggest that peroxisomal H_2_O_2_ could be involved in environmental change perception and acclimation through differential *PEX11* regulation and peroxisome proliferation. Plant peroxisome proliferation could be a protective response to ROS overflow in cell compartments due to highly efficient peroxisomal antioxidant defenses, as reported during protoplast transition from G0 to G1 (Tiew et al., 2015) and in response to salt stress in Arabidopsis and *Oryza sativa* by reducing both Na^+^ accumulation and oxidative stress (Cui et al., 2016b; Fahy et al., 2017). However, in quinoa plants, principal component analyses show a negative correlation of peroxisome abundance with yields in plants exposed to heat, drought or both (Hinojosa et al., 2019). Therefore, the capacity to maintain H_2_O_2_ homeostasis and peroxisome quality control/abundance could determine the success of plant adaptation to adverse conditions.

Box 2 Peroxisome biogenesis and proliferation

Plant peroxisome abundance is governed by (1) biogenesis, associated with physiological processes and division (fission) of a preexisting peroxisome, (2) proliferation, which is related to stress responses, and (3) pexophagy, a selective peroxisome degradation mechanism (Kao et al., 2018; Olmedilla and Sandalio, 2019). Proteins involved in peroxisome biogenesis and maintenance are called peroxins (PEXs; Kao et al., 2018). The import of peroxisomal membrane proteins (PMPs) in Arabidopsis involves peroxins PEX19, acting as the chaperone for PMPs, PEX3 acting as the membrane anchor for PEX19, and PEX16, which recruits PEX3 to the ER before the formation of pre-peroxisomes. Arabidopsis PEX16 also recruits PMPs to the ER in a PEX3/PEX19-independent manner (Pan et al., 2020). The import of peroxisomal matrix proteins containing the C-and N-terminal targeting signals PTS1 and PTS2, respectively, take place by the soluble receptors PEX5 (for PTS1) and PEX7 (for PTS2) in the cytosol (Baker et al., 2016; Reumann and Chowdhary, 2018). PEX5 is recycled from the peroxisomal matrix back to the cytosol by the ubiquitin conjugating enzyme PEX4 and its membrane anchor PEX22, three RING-type ubiquitin ligases, PEX2, PEX10 and PEX12, and two AAA ATPases, PEX1 and PEX6 (reviewed in Reumann and Bartel, 2016; Kao et al., 2018).

Peroxisomes proliferate through the division of preexistent peroxisomes, which involves peroxisome elongation regulated by PEX11, organelle constriction and fission, governed by dynamin-related proteins (DRP3A and DRP3B) and fission proteins (FIS1A and FIS 1B; Pan et al., 2020; Su et al., 2019). The PEX11 gene family in Arabidopsis contain five members: PEX11a, PEX11b, PEX11c, PEX11d and PEX11e, (Lingard and Trelease, 2006; Orth et al., 2007). FIS1A and FIS1B are shared by peroxisomes and mitochondria; DRP3A and DRP3B regulate peroxisomal and mitochondrial fission; and DRP5B is involved in peroxisome and chloroplast fission (Kao et al., 2018), indicating a highly coordinated regulation of organelles populations.

### ROS/RNS-dependent formation of peroxules

In vivo observation of plant tissues, with fluorescent proteins targeting peroxisomes, reveals the rapid formation of tubular peroxisome extensions called peroxules, induced in response to changes in ROS levels (Figure 3; Sinclair et al., 2009; Barton et al., 2013; Rodríguez-Serrano et al., 2016). Short periods of Cd exposure (15–30 min) induce peroxule formation, which is considerably reduced by H_2_O_2_ scavengers and in *rboh* mutants, suggesting regulation by external ROS (Rodríguez-Serrano et al., 2016). Confocal images show peroxule contacts with chloroplasts and mitochondria under Cd treatment and high light (Sinclair et al., 2009; Sandalio et al., 2013; Jaipargas et al., 2016; Rodríguez-Serrano et al., 2016). Peroxule formation in response to Cd and As is dependent upon PEX11a, while *pex11a* Arabidopsis mutants show altered ROS-dependent signaling networks (Rodríguez-Serrano et al., 2016). Peroxule production and peroxisome-dependent signaling are compromised in *nia1 nia2* Arabidopsis mutants, which have lower NO levels than wild-type plants in response to Cd treatment, demonstrating the important role of NO in peroxule formation (Terrón-Camero et al., 2020). This could be due to oxidative changes and *S*-nitrosylation patterns in the antioxidant system (Terrón-Camero et al., 2020), which affect cellular redox balance. PEX11a and peroxule formation therefore play a key role in regulating stress perception and rapid cell responses to environmental cues (Rodríguez-Serrano et al., 2016; Terrón-Camero et al., 2020; Figure 3). Given rapid peroxule induction and no significant changes in *PEX11a* expression in *nox1* mutants (Terrón-Camero et al., 2020), PEX11a can reasonably be assumed to be regulated by specific ROS- and NO-dependent PTMs. Activation of yeast peroxin Pex11p depends on redox changes in its cysteines (Knoblach and Rachubinski, 2010; Schrader et al., 2012).

**Figure 4:**
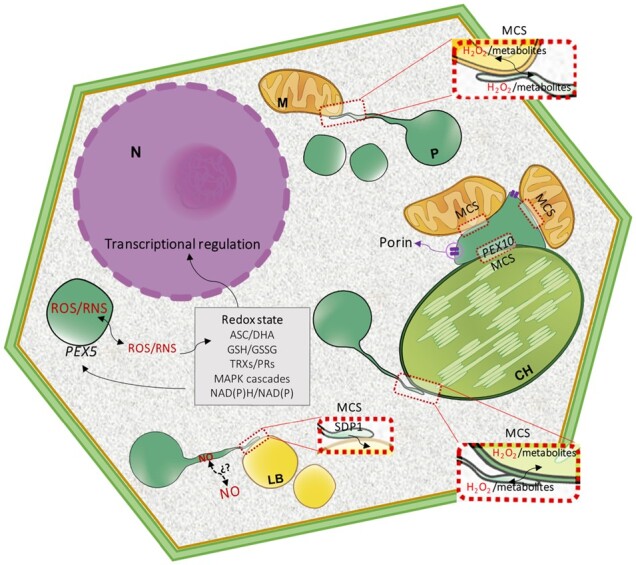
Redox-dependent interorganellar crosstalk. Peroxisomes (P) collaborate and communicate with other cellular organelles, mitochondria (M), and chloroplasts (CH) by exchanging molecules such as H_2_O_2_ and redox metabolites, as well as Ca^2+^ and proteins. These exchanges could take place through porins or MCSs. Peroxule formation contributes to ROS/RNS, metabolite, and protein exchanges such as the transfer of TAG lipase sugar-dependent 1 (SDP1) to lipid bodies (LB). ROS/RNS-dependent posttranslational modifications regulate peroxule formation, MCSs, interorganellar crosstalk, and signaling transduction. Peroxisomal ROS/RNS interferes with cytosolic redox state and signaling processes and vice versa; the cytosolic redox state regulates peroxisomal protein import by affecting the redox state of peroxin 5 (PEX5). The peroxisomal redox state can also regulate redox changes in the nucleus (N), chloroplasts, and mitochondria.

Although there is no direct evidence, peroxules could participate in the transfer of H_2_O_2_ and other metabolites to mitochondria and chloroplast (Figure 4). Stromules, which are dynamic structures similar to peroxules in chloroplasts, transfer H_2_O_2_ from chloroplasts to nuclei as part of a retrograde signaling process (Caplan et al., 2015; Kumar et al., 2018); however, to our knowledge, no connection between peroxules and nuclei has been established so far. Peroxules could also be involved in protein transport such as the transfer of the sugar-dependent 1 (SDP1) lipase from the peroxisomal membrane to the lipid body (Thazar-Poulot et al., 2015; Figure 4).

### Peroxisomal speed is regulated by ROS

Time course analyses of peroxisomes in response to Cd in Arabidopsis seedlings have highlighted a considerable increase in peroxisomal speed after 24 h of treatment which is regulated by ROS produced by RBOHs and Ca^2+^ ions (Figure 3; Rodríguez-Serrano et al., 2009, 2016). Increased peroxisomal movement could improve antioxidant defenses where Cd and other factors promote ROS accumulation and/or could aid signaling transduction and metabolite exchanges in different parts of the cell (Rodríguez-Serrano et al., 2009). Information on the role of peroxisomal motility under stress conditions is scarce; however, in myosin loss-of-function Arabidopsis mutants and in Arabidopsis treated with the herbicide 2,4-D, inhibition of organelle movement negatively affects plant growth (Rodríguez-Serrano et al., 2014; Ryan and Nebenführ, 2018). Oikawa et al. (2015) found that light-adapted Arabidopsis peroxisomes are much more mobile than dark-adapted peroxisomes (Oikawa et al., 2015) and, by using photorespiratory mutants *shmt1* (defective in serine hydroxymethyltransferase) and *ped2* (defective in PEX14), they concluded that both, peroxisome mobility and peroxisome–chloroplast interactions observed under light, are regulated by photosynthesis rather than by photoreceptors or photorespiration (Oikawa et al., 2015).

### Pexophagy and peroxisomal homeostasis are regulated by oxidative processes.

Excessive numbers of peroxisomes and those containing obsolete or dysfunctional proteins need to be eliminated to control cellular redox homeostasis. Some evidence shows that ROS and oxidative damage to peroxisomes regulate the degradation of oxidized whole peroxisomes by selective autophagy termed pexophagy (Figure 3; Shibata et al., 2013; Yoshimoto et al., 2014; Olmedilla and Sandalio, 2019). Autophagy-related genes (ATGs) regulate autophagy in all eukaryotic cells including those in plants (Avin-Wittenberg et al., 2018). During photomorphogenesis, several authors have reported pexophagy using Arabidopsis *atg* mutants (Farmer et al., 2013; Kim et al., 2013; Shibata et al. 2013); however, unlike in mammals and yeast, the mechanism in plants is not fully understood. Shibata et al. (2013) have observed high levels of oxidized CAT and clusters of peroxisomes in *atg* mutants. Peroxisomal clusters were also observed in H_2_O_2_-treated Arabidopsis plants (Yoshimoto et al., 2014) and in *atg5* and *atg7* Arabidopsis mutants exposed to Cd treatment where phagophore and peroxisome colocalization was observed (Calero-Muñoz et al., 2019). Some evidence shows the important role of oxidative processes in pexophagy induction: (1) ubiquitinated CAT is accumulated in Arabidopsis mutants defective in NBR1 (*nbr-1)*, a pexophagy adaptor (Zhou et al., 2013); (2) ATG8/CAT–CAT/NBR1 interactions have been observed in Arabidopsis plants exposed to Cd (Calero-Muñoz et al., 2019); (3) CAT activity is involved in starvation-induced pexophagy (Tyutereva et al., 2018); and (4) clustered peroxisomes in Arabidopsis *atg* mutants mainly accumulate in the aerial parts of plants, where oxidative metabolism is higher than in roots (Zhou et al., 2013; Yoshimoto et al., 2014). Glucose-mediated regulation of root meristem activity requires pexophagy to maintain ROS and auxin cellular homeostasis in Arabidopsis plants (Huang et al., 2019). The chaperone activity of peroxisomal protease LON2 negatively regulates pexophagy (Figure 3; Farmer et al., 2013; Young and Bartel, 2016). In plants, specific peroxisomal receptors have not been clearly identified, and the role of adaptors, such as NBR1-like proteins, which specifically interact with ubiquitinilated proteins, is under debate (Olmedilla and Sandalio, 2019; Young et al., 2019). The possibility of both NBR1-dependent and independent pexophagy cannot be ruled out. An alternative process independent of autophagy, induced under high CO_2_ and increased H_2_O_2_ conditions, involves chloroplast vesiculation (CV) proteins which interact with PEX11-1 in rice (Figure 3; Umnajkitikorn et al., 2020).

## Peroxisome crosstalk with other organelles

To optimize their multiple functions, peroxisomes collaborate and communicate with other cell organelles by exchanging substrates. Photorespiration is the best example of metabolic cellular interorganelle communication. However, peroxisomes are also cellular redox communication hubs, as well as guardians and modulators of H_2_O_2_ levels (Fransen and Lismont, 2019) given the following findings: (1) peroxisomes contain enzymes involved in producing and scavenging H_2_O_2_ and NO; (2) they contain proteins regulated by ROS- and NO-dependent PTMs and therefore act as ROS/NO sensors; (3) they regulate NAD(P)+/NAD(P)H, ascorbic (Asc)/dehydroascorbic acid (DHA) and GSH/GSSG pools; and (4) H_2_O_2_ and NO act as second messengers in a wide range of developmental, physiological, and stress processes (Fransen and Lismont, 2019; Sandalio et al., 2020).

There is an intimate relationship between the peroxisomal redox state and changes in the redox state of other organelles. In mammalian systems, H_2_O_2_ released from peroxisomes into the cytosol diffuses into mitochondria, oxidizing directly or indirectly cysteine residues of mitochondrial proteins (Lismont et al., 2019). Chlamydomonas mutants deficient in peroxisomal NAD^+^-dependent MDH2 show that MDH2 plays a key role in the reverse coupling of redox/H_2_O_2_ signals from peroxisomes to chloroplasts (Kong et al., 2018). Peroxisomal NAD(P)^+^/NAD(P)H pools in Arabidopsis regulate photosynthesis performance to meet the demand for reducing equivalents under fluctuating light (Li et al., 2019). Peroxisomal basal H_2_O_2_ levels greatly affect antioxidative defense regulation in cytosol and chloroplasts, as observed in peroxisomal *apx4* knockdown rice plants (Sousa et al., 2018). The inhibition of CAT activity in *apx4* Arabidopsis mutants significantly affected networks involved in photosynthetic performance under adverse conditions promoting oxidative stress and favoring antioxidant enzyme accumulation in cytosol and chloroplasts (Sousa et al., 2018).

Despite the central role of H_2_O_2_ in peroxisome metabolism and cell functionality, no peroxiporin-like proteins have been identified in the peroxisomal membrane. Although porins are present in plant peroxisomes (Reumann et al., 1997; Corpas et al., 2000; Figure 4), their role in H_2_O_2_ permeability remains unclear. In yeast, Pex11A, Pex11B, and Pex11G have been reported to facilitate the permeation of molecules up to 400 Da (Mindthoff et al., 2016) and could be candidates to diffuse H_2_O_2_ through the peroxisomal membrane. However, recently, Lismont et al. (2019) provided evidence that neither the porin PXMP2 nor PEX11B is essential for H_2_O_2_ permeation across the peroxisomal membrane. Throughout membrane contact sites (MCSs), ROS accumulation could directly facilitate interorganelle signal transmission using as-yet-unknown ROS transporters (Figure 4; Yoboue et al., 2018). Electron microscopy images of leaf cells show physical contact between peroxisome and chloroplasts and, interestingly, H_2_O_2_ accumulation inside peroxisomes in the contact site with chloroplasts and vacuoles, suggesting a relationship between ROS accumulation and organelle communication (Romero-Puertas et al., 2004). Using femtosecond laser and optic tweezer techniques, tethering between the chloroplast and peroxisomes has been demonstrated (Oikawa et al., 2015; Gao et al., 2016). The area of peroxisomes interacting with chloroplasts increases under light conditions, whereas, in the dark, peroxisomes lost their connection with chloroplasts (Oikawa et al., 2015). The PEX10 Zn RING finger interacts with the chloroplast envelope’s outer membrane, which is necessary for full photorespiration functionality and could be a candidate for MCSs in plant peroxisomes (Schumann et al., 2007; Figure 4). Although the role of PTMs in regulating protein–protein interactions at interorganelle contact sites remains unexplored, it is reasonable to assume that tethering is regulated by specific PTMs.

The translocation of peroxisomal proteins to other cell compartments is part of interorganelle communication and signaling, although the mechanism(s) by which this occurs is still unknown. Thus, CAT interacts with nonperoxisomal proteins including cytosolic calcium-dependent kinase CDPK8 (Zou et al., 2015), plasma membrane-associated calcium-dependent kinase OsCPK10 (Bundó and Coca, 2017), cytosolic salt overly sensitive 2 (SOS2; Verslues et al., 2007), lesion simulating disease1 (LSD1; Li et al., 2013), receptor-like cytoplasmic kinase STRK1 (Zhou et al., 2018), chloroplast/cytosolic nucleoside diphosphate kinase 2 (NDPK2), no catalase activity 1 (NCA1; Hackenberg et al., 2013; Li et al., 2015), and nucleoredoxin 1 (NRX1; Kneeshaw et al., 2017) in addition to GSNOR (mentioned above), which are all integral stress-signaling proteins. It is unclear whether CAT is translocated from peroxisomes by the ER-associated degradation (ERAD)-like system involved in the export of PEX5 from the peroxisome membrane and export of matrix peroxisomal proteins to be degraded (Lingard et al., 2009) or is retained in the cytosol under oxidative conditions as in the case of mammalian cells (Walton et al., 2017). Walton et al. (2017) have reported that Cys-11 of human PEX5 acts as a redox switch that modulates the import of peroxisomal matrix proteins such as CAT. Under oxidative stress conditions, CAT is retained in the cytosol where it can protect against H_2_O_2_-mediated redox changes and reinforce cellular defenses to prevent oxidative damage out of peroxisomes (Walton et al., 2017). Oxidative and nitrosative stress could enable swift control of CAT localization in compartments, thus helping to regulate redox signaling pathways.

In the case of dual-targeted OPPP enzymes, targeting decisions appear to depend on the cytosolic redox state. This has been suggested in relation to Arabidopsis G6PD1/G6PD4 (Meyer et al., 2011), PGL3 (Hölscher et al., 2014), and PGD2 upon interaction with nonperoxisomal isoforms PGD1 or PGD3, which retain heteromeric enzymes in the cytosol (Lutterbey and von Schaewen, 2016). NADPH-oxidase and peroxisomal AtPAO3 cross-talk to balance intracellular O_2_^.−^/H_2_O_2_, which in turn, affect the cyt-c/AOX pathways in mitochondria and regulates pollen tube elongation (Wu et al., 2010). As catalase (*cat2*)-deficient Arabidopsis mutants show upregulation of ASC-GSH components in the cytosol (Mhamdi et al., 2010), peroxisomes can interfere with cytosolic redox state. Cytosolic redox changes, in turn, impact the dual targeting of 6-phosphogluconolactonase 3 (PGL3) of chloroplasts and peroxisomes in Arabidopsis leaves, a process requiring thioredoxin m2 (Trxm2) in the cytosol (Hölscher et al., 2014).

## Future perspectives

Many challenges in peroxisome redox biology remain to be addressed (Outstanding questions). One of them is to determine the nature of proteins involved in peroxisomal NO production. We need to amplify our limited knowledge of the mechanisms underlying NO regulation of peroxisome dynamics, metabolism, and signaling, together with NO and ROS crosstalk with hormones, such as JA. Additional analyses of the interplay and hierarchy of ROS-, NO-dependent, and other peroxisomal PTMs such as phosphorylation and persulfidation are required. Peroxisome-dependent regulatory components also need to be characterized by analyzing gene network structures and by identifying downstream responses induced by peroxisomal ROS and NO. The components of contact sites and the factors involved in peroxule production, as well as the regulatory role of ROS and NO in both these areas, also need to be determined. Analysis of tethering techniques, specific fluorescence proteins, and ROS mutants, combined with meta-analyses of organelle proteome datasets, should provide a better understanding of peroxisomal dynamics and interorganelle interactions. Finally, identification of pexophagy receptors and adaptors and their regulation by ROS, NO, and S_2_H should enable us to integrate biochemical processes and organelle dynamics into our understanding of cellular regulatory systems.

**OUTSTANDING QUESTIONS**

- Do carbonylated and nitrated peroxisomal proteins act as signalling messengers?
- Does CAT3 transnitrosylate other proteins in addition to GSNOR? Are other transnitrosylases present in peroxisomes?
- Is *S*-nitrosylation involved in retrotranslocation of peroxisomal proteins to the cytosol?
- Which pexophagy receptors and adaptors are involved and what role do ROS and NO play in identifying peroxisomes for degradation?
- How do cells balance peroxule production, peroxisome proliferation and changes in peroxisomal speed in response to changes in their environment?
- Which ROS/redox sensor(s) is (are) present in the peroxisomal membrane?

## Notes

L.M.S. conceived the original review focus and wrote the article with input and critical discussion from all the authors; M.A.P.-V. and E.M.-M. edited the figures. All authors read and approved the content of the manuscript. L.M.S. has agreed to act as the author responsible for contacts and correspondence.

The author responsible for distribution of materials integral to the findings presented in this article in accordance with the policy described in the Instructions for Authors (https://academic.oup.com/plphys/pages/general-instructions) is: Luisa M. Sandalio (luisamaria.sandalio@eez.csic.es).

## References

[kiab060-B1] Agurla S , Gayatri G, Raghavendra AS (2018) Polyamines increase nitric oxide and reactive oxygen species in guard cells of *Arabidopsis thaliana* during stomatal closure. Protoplasma 255: 153–162 28699025 10.1007/s00709-017-1139-3

[kiab060-B2] Alamillo JM , García-Olmedo F (2001) Effects of urate, a natural inhibitor of peroxynitrite-mediated toxicity, in the response of *Arabidopsis thaliana* to the bacterial pathogen *Pseudomonas syringae*. Plant J 25: 529–540 11309143 10.1046/j.1365-313x.2001.00984.x

[kiab060-B3] Antonenkov VD , Grunau S, Ohlmeier S, Hiltunen JK (2010) Peroxisomes are oxidative organelles. Antioxid Redox Signal 13: 525–537 19958170 10.1089/ars.2009.2996

[kiab060-B4] Avin-Wittenberg T , Baluška F, Bozhkov PV, Elander PH, Fernie AR, Galili G, Hassan A, Hofius D, Isono E, Le Bars R, et al (2018) Autophagy-related approaches for improving nutrient use efficiency and crop yield protection. J Exp Bot 69: 1335–1353 29474677 10.1093/jxb/ery069

[kiab060-B5] Baker A , Hogg TL, Warriner SL (2016) Peroxisome protein import: a complex journey. Biochem Soc Trans 44: 783–789 27284042 10.1042/BST20160036 PMC4900764

[kiab060-B6] Barroso JB , Corpas FJ, Carreras A, Sandalio LM, Valderrama R, Palma M, Lupiáñez JA, del Río LA (1999) Localization of nitric-oxide synthase in plant peroxisomes. Int J Biol Chem 274: 36729–36733 10.1074/jbc.274.51.36729 10593979

[kiab060-B7] Barton K , Mathur N, Mathur J (2013) Simultaneous live-imaging of peroxisomes and the ER in plant cells suggests contiguity but no luminal continuity between the two organelles. Front Physiol 4: 1–12 23898304 10.3389/fphys.2013.00196 PMC3721060

[kiab060-B8] Bratt A , Rosenwasser S, Meyer A, Fluhr R (2016) Organelle redox autonomy during environmental stress. Plant Cell Environ 39: 1909–1919 27037976 10.1111/pce.12746

[kiab060-B9] Bundó M , Coca M (2017) Calcium-dependent protein kinase OsCPK10 mediates both drought tolerance and blast disease resistance in rice plants. J Exp Bot 68: 2963–2975 28472292 10.1093/jxb/erx145 PMC5853374

[kiab060-B10] Byrne RS , Ha R, Mendel RR, Hille R (2009) Oxidative half-reaction of *Arabidopsis thaliana* sulfite oxidase generation of superoxide by a peroxisomal enzyme. Int J Bio Chem 284: 35479–35484 10.1074/jbc.M109.067355 PMC2790977 19875441

[kiab060-B11] Calero-Muñoz N , Expósito-Rodríguez M, Collado-Arenal AM, Rodríguez-Serrano M, Laureano-Marín AM, Santamaría ME, Gotor C, Díaz I, Mullineaux PM, Romero-Puertas MC, et al (2019) Cadmium induces reactive oxygen species-dependent pexophagy in Arabidopsis leaves. Plant Cell Environ 42: 2696–2714 31152467 10.1111/pce.13597

[kiab060-B12] Caplan JL , Kumar AS,, Park E, Padmanabhan MS, Hoban K, Modla S, Czymmek K, Dinesh-Kumar SP (2015) Chloroplast stromules function during innate immunity. Dev Cell 34: 45–57 26120031 10.1016/j.devcel.2015.05.011 PMC4596411

[kiab060-B13] Castillo MC , Sandalio LM, del Río LA, León J (2008) Peroxisome proliferation, wound-activated responses and expression of peroxisome-associated genes are cross-regulated but uncoupled in *Arabidopsis thaliana*. Plant Cell Environ 31: 492–505 18194426 10.1111/j.1365-3040.2008.01780.x

[kiab060-B14] Chaki M , Álvarez de Morales P, Ruiz C, Begara-Morales JC, Barroso JB, Corpas FJ, Palma JM (2015) Ripening of pepper (*Capsicum annuum*) fruit is characterized by an enhancement of protein tyrosine nitration. Ann Bot 116: 637–47 25814060 10.1093/aob/mcv016 PMC4577987

[kiab060-B15] Chaouch S , Queval G, Vanderauwera S, Mhamdi A, Vandorpe M, Langlois-Meurinne M, Van Breusegem F, Saindrenan P, Noctor G (2010) Peroxisomal hydrogen peroxide is coupled to biotic defense responses by ISOCHORISMATE SYNTHASE1 in a daylength-related manner. Plant Physiol 153: 1692–1705 20543092 10.1104/pp.110.153957 PMC2923881

[kiab060-B16] Chen L , Wu R, Feng J, Feng T, Wang C, Hu J, Zhan N, Li Y, Ma X, Ren B, Zhang J, Song C-P, et al (2020) Transnitrosylation mediated by the non-canonical catalase ROG1 regulates nitric oxide signaling in plants. Dev Cell 53: 444–457 32330424 10.1016/j.devcel.2020.03.020

[kiab060-B17] Corpas FJ, , Barroso JB (2014) Peroxynitrite (${\text{ONOO}}_{}^{-}$) is endogenously produced in Arabidopsis peroxisomes and is overproduced under cadmium stress. Ann Bot 113: 87–96 24232384 10.1093/aob/mct260 PMC3864731

[kiab060-B18] Corpas FJ, , Barroso JB, Sandalio LM, Palma M, Lupiáñez JA, del Río LA (1999) Characterization and activity regulation during natural senescence. Plant Physiol 121: 921–928 10557241 10.1104/pp.121.3.921 PMC59455

[kiab060-B19] Corpas FJ , Sandalio LM, Brown MJ, del Río LA, Trelease RN (2000) Identification of porin-like polypeptide(s) in the boundary membrane of oilseed glyoxysomes. Plant Cell Physiol 41: 1218–1228 11092906 10.1093/pcp/pcd054

[kiab060-B20] Costa A , Drago I, Behera S, Zottini M, Pizzo P, Schroeder JI, Pozzan T, Lo Schiavo F (2010) H_2_O_2_ in plant peroxisomes: an in vivo analysis uncovers a Ca^2+^-dependent scavenging system. Plant J 62: 760–72 20230493 10.1111/j.1365-313X.2010.04190.x PMC2884081

[kiab060-B21] Cousins AB , Pracharoenwattana I, Zhou W, Smith SM, Badger MR (2008) Peroxisomal malate dehydrogenase is not essential for photorespiration in Arabidopsis but its absence causes an increase in the stoichiometry of photorespiratory CO_2 _release. Plant Physiol 148: 786–795 18685043 10.1104/pp.108.122622 PMC2556826

[kiab060-B22] Cui L , Lu Y, Li Y, Yang C, Peng X, Lines TR (2016a) Overexpression of glycolate oxidase confers improved photosynthesis under high light and high temperature in rice. Front Plant Sci 7: 1165 27540387 10.3389/fpls.2016.01165 PMC4972838

[kiab060-B23] Cui P , Liu H, Islam F, Li L, Farooq MA, Ruan S, Zhou W (2016b) OsPEX11, a peroxisomal biogenesis factor 11, contributes to salt stress tolerance in *Oryza sativa*. Front Plant Sci 7: 1–11 27695459 10.3389/fpls.2016.01357 PMC5024708

[kiab060-B24] Del Río LA , Corpas FJ, Sandalio LM, Palma JM, Barroso JB (2003) Plant peroxisomes, reactive oxygen metabolism and nitric oxide. IUBMB Life 55: 71–81 12749689 10.1080/1521654031000094694

[kiab060-B25] Duan G , Walther D (2015) The roles of post-translational modifications in the context of protein interaction networks. PLoS Comput Biol 11: 1–23 10.1371/journal.pcbi.1004049 PMC4333291 25692714

[kiab060-B26] Eastmond PJ (2007) MONODEHYROASCORBATE REDUCTASE4 is required for seed storage oil hydrolysis and postgerminative growth in Arabidopsis. Plant Cell 19: 1376–1387 17449810 10.1105/tpc.106.043992 PMC1913749

[kiab060-B27] Ebeed HT , Stevenson SR, Cuming AC, Baker A (2018) Conserved and differential transcriptional responses of peroxisome associated pathways to drought, dehydration and ABA. J Exp Bot 69: 4971–4985 30032264 10.1093/jxb/ery266 PMC6137984

[kiab060-B28] Fahy D , Sanad MNME, Duscha K, Lyons M, Liu F, Bozhkov P, Kunz HH,, Hu J, Neuhaus HE, Steel PG, et al (2017) Impact of salt stress, cell death, and autophagy on peroxisomes: quantitative and morphological analyses using small fluorescent probe N-BODIPY. Sci Rep 7: 1–17 28145408 10.1038/srep39069 PMC5286434

[kiab060-B29] Farmer LM , Rinaldi MA, Young PG, Danan CH, Burkhart SE, Bartel B (2013) Disrupting autophagy restores peroxisome function to an Arabidopsis lon2 mutant and reveals a role for the LON2 protease in peroxisomal matrix protein degradation. Plant Cell 25: 4085–4100 24179123 10.1105/tpc.113.113407 PMC3877801

[kiab060-B30] Foyer CH , Baker A, Wright M, Sparkes IA, Mhamdi A, Schippers JHM, Van Breusegem F (2020) On the move: redox-dependent protein relocation in plants. J Exp Bot 71: 620–631 31421053 10.1093/jxb/erz330

[kiab060-B31] Foyer CH , Bloom AJ, Queval G, Noctor G (2009) Photorespiratory metabolism: genes, mutants, energetics and redox signaling. Ann rev Plant Bio 60: 455–484 19575589 10.1146/annurev.arplant.043008.091948

[kiab060-B32] Foyer CH , Noctor G (2003) Redox sensing and signalling associated with reactive oxygen in chloroplasts, peroxisomes and mitochondria. Physiol Plant 119: 355–364

[kiab060-B33] Foyer CH , Noctor G (2016) Stress-triggered redox signalling: what’s in pROSpect? Front Plant Sci 39: 951–964 10.1111/pce.12621 26264148

[kiab060-B34] Foyer CH , Ruban A V, Noctor G (2017) Viewing oxidative stress through the lens of oxidative signalling rather than damage. Biochem J 474: 877–883 28270560 10.1042/BCJ20160814 PMC5469280

[kiab060-B35] Fransen M , Lismont C (2019) Redox signaling from and to peroxisomes: Progress, challenges, and prospects. Antioxid Redox Signal 30: 95–112 29433327 10.1089/ars.2018.7515

[kiab060-B36] Gabaldón T (2018) Evolution of the peroxisomal proteome. Subcell Biochem 89: 221–233 30378025 10.1007/978-981-13-2233-4_9

[kiab060-B37] Gao H , Metz J, Teanby NA, Ward AD, Botchway SW, Coles B, Pollard MR, Sparkes I (2016) In vivo quantification of peroxisome tethering to chloroplasts in tobacco epidermal cells using optical tweezers. Plant Physiol 170: 263–72 26518344 10.1104/pp.15.01529 PMC4704594

[kiab060-B38] Gebicka L , Didik J (2009) Catalytic scavenging of peroxynitrite by catalase. J Inorg Biochem 103: 1375–1379 19709751 10.1016/j.jinorgbio.2009.07.011

[kiab060-B39] Gechev TS , Minkov IN, Hille J (2005) Hydrogen peroxide-induced cell death in Arabidopsis: transcriptional and mutant analysis reveals a role of an oxoglutarate-dependent dioxygenase gene in the cell death process. IUBMB Life 57: 181–188 16036580 10.1080/15216540500090793

[kiab060-B40] Goyer A , Johnson TL, Olsen LJ, Collakova E, Shachar-Hill Y, Rhodes D, Hanson AD (2004) Characterization and metabolic function of a peroxisomal sarcosine and pipecolate oxidase from Arabidopsis. J Biol Chem 279: 16947–16953 14766747 10.1074/jbc.M400071200

[kiab060-B41] Gupta DK , Pena LB, Hernández A, Inouhe M, Sandalio LM (2017) NADPH oxidases differentially regulate ROS metabolism and nutrient uptake under cadmium toxicity. Plant Cell Environ 40: 509–526 26765289 10.1111/pce.12711

[kiab060-B42] Hackenberg T , Juul T, Auzina A, Gwizdz S, Małolepszy A, Van Der Kelen K, Dam S, Bressendorff S, Lorentzen A, Roepstorff P, et al (2013) Catalase and NO CATALASE ACTIVITY1 promote autophagy-dependent cell death in Arabidopsis. Plant Cell 25: 4616–4626 24285797 10.1105/tpc.113.117192 PMC3875739

[kiab060-B43] Hashiguchi A , Komatsu S (2016) Impact of post-translational modifications of crop proteins under abiotic stress. Proteomes 4: 42 10.3390/proteomes4040042 PMC5260974 28248251

[kiab060-B44] Hinojosa L , Sanad MNME, Jarvis DE, Steel P, Murphy K, Smertenko A (2019) Impact of heat and drought stress on peroxisome proliferation in quinoa. Plant J 99: 1144–1158 31108001 10.1111/tpj.14411

[kiab060-B45] Hodges M , Dellero Y, Keech O, Betti M, Raghavendra AS, Sage R, Zhu X, Allen DK, Weber APM (2016) Perspectives for a better understanding of the metabolic integration of photorespiration within a complex plant primary metabolism network. J Exp Bot 67: 3015–3026 27053720 10.1093/jxb/erw145

[kiab060-B46] Hölscher C , Meyer T, Von Schaewen A (2014) Dual-targeting of Arabidopsis 6-phosphogluconolactonase 3 (PGL3) to chloroplasts and peroxisomes involves interaction with Trx m2 in the cytosol. Mol Plant 7: 252–255 24008768 10.1093/mp/sst126

[kiab060-B47] Holzmeister C , Gaupels F, Geerlof A, Sarioglu H, Sattler M, Durner J, Lindermayr C (2015) Differential inhibition of Arabidopsis superoxide dismutases by peroxynitrite-mediated tyrosine nitration. J Exp Bot 66: 989–999 25428993 10.1093/jxb/eru458 PMC4321555

[kiab060-B48] Hooper DC , Scott GS, Zborek A, Mikheeva T, Kean RB, Koprowski H, Spitsin SV (2000) Uric acid, a peroxynitrite scavenger, inhibits CNS inflammation, blood-CNS barrier permeability changes, and tissue damage in a mouse model of multiple sclerosis. FASEB J 14: 691–698 10744626 10.1096/fasebj.14.5.691

[kiab060-B49] Hou Q , Bartels D (2015) Comparative study of the aldehyde dehydrogenase (ALDH) gene superfamily in the glycophyte *Arabidopsis thaliana* and *Eutrema halophytes*. Ann Bot 22: 465–479 10.1093/aob/mcu152 PMC4332599 25085467

[kiab060-B50] Huang J , Willems P, Breusegem F, Van Messens J (2018) Pathways crossing mammalian and plant sulfenomic landscapes. Free Radic Biol Med 122: 193–201 29476921 10.1016/j.freeradbiomed.2018.02.012

[kiab060-B51] Huang L , Yu LJ, Zhang X, Fan B, Wang FZ, Dai YS, Qi H, Zhou Y, Xie LJ, Xiao S (2019) Autophagy regulates glucose-mediated root meristem activity by modulating ROS production in Arabidopsis. Autophagy 15: 407–422 30208757 10.1080/15548627.2018.1520547 PMC6351127

[kiab060-B52] Inaba J , Kim BM, Shimura H, Masuta C (2011) Virus-induced necrosis is a consequence of direct protein-protein interaction between a viral RNA-silencing suppressor and a host catalase. Plant Physiol 156: 2026–2036 21622812 10.1104/pp.111.180042 PMC3149961

[kiab060-B53] Jaipargas EA , Mathur N, Daher FB, Wasteneys GO, Mathur J (2016) High light intensity leads to increased peroxule-mitochondria interactions in plants. Front Cell Dev Biol 4: 1–11 26870732 10.3389/fcell.2016.00006 PMC4740372

[kiab060-B54] Jones DP , Go YM (2010) Redox compartmentalization and cellular stress. Diabetes Obes Metab 12: 116–125 21029308 10.1111/j.1463-1326.2010.01266.x PMC3052693

[kiab060-B55] Kao Y , González KL, Bartel B (2018) Peroxisome function, biogenesis, and dynamics in plants. Plant Physiol 176: 162–177 29021223 10.1104/pp.17.01050 PMC5761812

[kiab060-B56] Kaur N , Reumann S, Hu J (2009) Peroxisome biogenesis and function. Arabidopsis Book 7: e0123 22303249 10.1199/tab.0123 PMC3243405

[kiab060-B57] Kim J , Lee H, Lee HN, Kim SH, Shin KD, Chung T (2013) Autophagy-related proteins are required for degradation of peroxisomes in Arabidopsis hypocotyls during seedling growth. Plant Cell 25: 4956–4966 24368791 10.1105/tpc.113.117960 PMC3903998

[kiab060-B58] Kneeshaw S , Keyani R, Delorme-Hinoux V, Imrie L, Loake GJ, Le Bihan T, Reichheld JP, Spoel SH (2017) Nucleoredoxin guards against oxidative stress by protecting antioxidant enzymes. Proc Natl Acad Sci USA 114: 8414–8419 28724723 10.1073/pnas.1703344114 PMC5547615

[kiab060-B59] Knoblach B , Rachubinski RA (2010) Phosphorylation-dependent activation of peroxisome proliferator protein PEX11 controls peroxisome abundance. J Biol Chem 285: 6670–6680 20028986 10.1074/jbc.M109.094805 PMC2825462

[kiab060-B60] Kong F , Burlacot A, Liang Y, Légeret B, Alseekh S, Brotman Y, Fernie AR, Krieger-Liszkay A, Beisson F, Peltier G, et al (2018) Interorganelle communication: peroxisomal MALATE DEHYDROGENASE2 connects lipid catabolism to photosynthesis through redox coupling in Chlamydomonas. Plant Cell 30: 1824–1847 29997239 10.1105/tpc.18.00361 PMC6139685

[kiab060-B61] König J , Muthuramalingam M, Dietz KJ (2012) Mechanisms and dynamics in the thiol/disulfide redox regulatory network: transmitters, sensors and targets. Curr Opin Plant Biol 15: 261–268 22226570 10.1016/j.pbi.2011.12.002

[kiab060-B62] Koo AJK , Chung HS, Kobayashi Y, Howe GA (2006) Identification of a peroxisomal acyl-activating enzyme involved in the biosynthesis of jasmonic acid in Arabidopsis. Int J Biol Chem 281: 33511–33520 10.1074/jbc.M607854200 16963437

[kiab060-B63] Kumar AS, , Park E, Nedo A, Alqarni A, Ren L, Hoban K, Modla S, McDonald JH, Kambhamettu C, Dinesh-Kumar SP, et al (2018) Stromule extension along microtubules coordinated with actin-mediated anchoring guides perinuclear chloroplast movement during innate immunity. Elife 7: 1–33 10.7554/eLife.23625 PMC5815851 29338837

[kiab060-B64] Lansing H , Doering L (2020) Analysis of potential redundancy among Arabidopsis 6-phosphogluconolactonase isoforms in peroxisomes. J Exp Bot 71: 823–836 31641750 10.1093/jxb/erz473

[kiab060-B65] León J , Broseta AC (2020) Present knowledge and controversies, deficiencies, and misconceptions on nitric oxide synthesis, sensing, and signaling in plants. Plant Cell Environ 43: 1–15 10.1111/pce.13617 31323702

[kiab060-B66] Li J , Hu J (2015) Using co-expression analysis and stress-based screens to uncover Arabidopsis peroxisomal proteins involved in drought response. PLoS One 10: 1–13 10.1371/journal.pone.0137762 PMC4569587 26368942

[kiab060-B67] Li J , Liu J, Wang G, Cha JY, Li G, Chen S, Li Z, Guo J, Zhang C, Yang Y, et al (2015) A chaperone function of NO CATALASE ACTIVITY1 Is required to maintain catalase activity and for multiple stress responses in Arabidopsis. Plant Cell 27: 908–925 25700484 10.1105/tpc.114.135095 PMC4558663

[kiab060-B68] Li J , Tietz S, Cruz JA, Strand DD, Xu Y, Chen J, Kramer DM, Hu J (2019) Photometric screens identified Arabidopsis peroxisome proteins that impact photosynthesis under dynamic light conditions. Plant J 97: 460–474 30350901 10.1111/tpj.14134

[kiab060-B69] Li Y , Chen L, Mu J, Zuo J (2013) LESION SIMULATING DISEASE1 interacts with catalases to regulate hypersensitive cell death in Arabidopsis. Plant Physiol 163: 1059–1070 23958864 10.1104/pp.113.225805 PMC3793025

[kiab060-B70] Lingard MJ , Monroe-Augustus M, Bartel B (2009) Peroxisome-associated matrix protein degradation in Arabidopsis. Proc Natl Acad Sci USA 106: 4561–4566 19246395 10.1073/pnas.0811329106 PMC2657447

[kiab060-B71] Lingard MJ , Trelease RN (2006) Five Arabidopsis peroxin 11 homologs individually promote peroxisome elongation, duplication or aggregation. J Cell Sci 119: 1961–1972 16636080 10.1242/jcs.02904

[kiab060-B72] Lingner T , Kataya AR, Antonicelli GE, Benichou A, Nilssen K, Chen X, Siemsen T, Morgenstern B, Meinicke P, Reumann S (2011) Identification of novel plant peroxisomal targeting signals by a combination of machine learning methods and in vivo subcellular targeting analyses. Plant Cell 23: 1556–1572 21487095 10.1105/tpc.111.084095 PMC3101550

[kiab060-B73] Lismont C , Koster J, Provost S, Baes M, Van Veldhoven PP, Waterham HR, Fransen M (2019) Deciphering the potential involvement of PXMP2 and PEX11B in hydrogen peroxide permeation across the peroxisomal membrane reveals a role for PEX11B in protein sorting. Biochim Biophys Acta Biomembr 1861: 182991 31129117 10.1016/j.bbamem.2019.05.013

[kiab060-B74] López-Huertas E , Charlton WL, Johnson B, Graham IA, Baker A (2000) Stress induces peroxisome biogenesis genes. EMBO J 19: 6770–6777 11118212 10.1093/emboj/19.24.6770 PMC305880

[kiab060-B75] Lozano-Juste J , Colom-moreno R, Valencia D, Ed CPI, De Vera C (2011) In vivo protein tyrosine nitration in *Arabidopsis thaliana*. J Exp Bot 62: 3501–3517 21378116 10.1093/jxb/err042 PMC3130175

[kiab060-B76] Lutterbey MC , von Schaewen A (2016) Analysis of homo- and hetero-dimerization among the three 6-phosphogluconate dehydrogenase isoforms of Arabidopsis. Plant Signal Behav 11: 1–4 10.1080/15592324.2016.1207034 PMC5117088 27366940

[kiab060-B77] Martínez-Ruiz A , Cadenas S, Lamas S (2011) Nitric oxide signaling: classical, less classical, and nonclassical mechanisms. Free Radic Biol Med 51: 17–29 21549190 10.1016/j.freeradbiomed.2011.04.010

[kiab060-B78] Meyer T , Hölscher C, Schwöppe C, Von Schaewen A (2011) Alternative targeting of Arabidopsis plastidic glucose-6-phosphate dehydrogenase G6PD1 involves cysteine-dependent interaction with G6PD4 in the cytosol. Plant J 66: 745–758 21309870 10.1111/j.1365-313X.2011.04535.x

[kiab060-B79] Mhamdi A , Noctor G, Baker A (2012) Plant catalases: peroxisomal redox guardians. Arch Biochem Biophys 525: 181–194 22546508 10.1016/j.abb.2012.04.015

[kiab060-B80] Mhamdi A , Queval G, Chaouch S, Vanderauwera S, Van Breusegem F, Noctor G (2010) Catalase function in plants: a focus on Arabidopsis mutants as stress-mimic models. J Exp Bot 61: 4197–220 20876333 10.1093/jxb/erq282

[kiab060-B81] Mindthoff S , Grunau S, Steinfort LL, Girzalsky W, Hiltunen JK, Erdmann R, Antonenkov VD (2016) Peroxisomal Pex11 is a pore-forming protein homologous to TRPM channels. Biochim Biophys Acta 1863: 271–283 26597702 10.1016/j.bbamcr.2015.11.013

[kiab060-B82] Mitsuya S , El-Shami M, Sparkes I, Charlton WL, Lousa CDM, Johnson B, Baker A (2010) Salt stress causes peroxisome proliferation, but inducing peroxisome proliferation does not improve NaCl tolerance in *Arabidopsis thaliana*. PLoS ONE 5: e9408 20195524 10.1371/journal.pone.0009408 PMC2827565

[kiab060-B83] Mittler R (2017) ROS are good. Trends Plant Sci 22: 11–19 27666517 10.1016/j.tplants.2016.08.002

[kiab060-B84] Møller IM , Sweetlove LJ (2010) ROS signalling-specificity is required. Trends Plant Sci 15: 370–374 20605736 10.1016/j.tplants.2010.04.008

[kiab060-B85] Mor A , Koh E, Weiner L, Rosenwasser S, Sibony-benyamini H, Fluhr R (2014) Singlet oxygen signatures are detected independent of light or chloroplasts in response to multiple stresses. Plant Physiol 165: 249–261 24599491 10.1104/pp.114.236380 PMC4012584

[kiab060-B86] Murota K , Shimura H, Takeshita M, Masuta C (2017) Interaction between cucumber mosaic virus 2b protein and plant catalase induces a specific necrosis in association with proteasome activity. Plant Cell Rep 36: 37–47 27659495 10.1007/s00299-016-2055-2 PMC5206265

[kiab060-B87] Nila AG , Sandalio LM, López MG, Gómez M, del Río LA,, Gómez-Lim MA (2006) Expression of a peroxisome proliferator-activated receptor gene (xPPARα) from *Xenopus laevis* in tobacco (*Nicotiana tabacum*) plants. Planta 224: 569–581 16738865 10.1007/s00425-006-0246-8

[kiab060-B88] Noctor G , Foyer CH (2016). Intracellular redox compartmentation and ROS-related communication in regulation and signaling. Plant Physiol. 171: 1581–1592 27208308 10.1104/pp.16.00346 PMC4936564

[kiab060-B89] Noctor G , Reichheld J, Foyer CH (2018) ROS-related redox regulation and signaling in plants. Semin Cell Dev Biol 80: 3–12 28733165 10.1016/j.semcdb.2017.07.013

[kiab060-B90] Oikawa K , Matsunaga S, Mano S, Kondo M, Yamada K, Hayashi M, Kagawa T, Kadota A, Sakamoto W, Higashi S, et al (2015) Physical interaction between peroxisomes and chloroplasts elucidated by *in situ* laser analysis. Nat Plants 1: 15035 27247035 10.1038/nplants.2015.35

[kiab060-B91] Oksanen E , Häikiö E, Sober J, Karnosky DF (2004) Ozone-induced H_2_O_2_ accumulation in field-grown aspen and birch is linked to foliar ultrastructure and peroxisomal activity. New Phytol 161: 791–799 33873720 10.1111/j.1469-8137.2003.00981.x

[kiab060-B92] Olmedilla A , Sandalio LM (2019) Selective autophagy of peroxisomes in plants: from housekeeping to development and stress responses. Front Plant Sci 10: 1–7 31555306 10.3389/fpls.2019.01021 PMC6722239

[kiab060-B93] Ortega-Galisteo AP , Rodríguez-Serrano M, Pazmiño DM, Gupta DK, Sandalio LM, Romero-Puertas MC (2012) S-Nitrosylated proteins in pea (*Pisum sativum L*.) leaf peroxisomes: changes under abiotic stress. J Exp Bot 63: 2089–2103 22213812 10.1093/jxb/err414 PMC3295397

[kiab060-B94] Orth T , Reumann S, Zhang X, Fan J, Wenzel D, Quan S, Hu J (2007) The PEROXIN11 protein family controls peroxisome proliferation in Arabidopsis. Plant Cell 19: 333–350 17220199 10.1105/tpc.106.045831 PMC1820951

[kiab060-B95] Pan R , Hu J (2018) Proteome of plant peroxisomes. Subcell Biochem 89: 3–45 30378017 10.1007/978-981-13-2233-4_1

[kiab060-B96] Pan R , Liu J, Wang S, Hu J (2020) Peroxisomes: versatile organelles with diverse roles in plants. New Phytol 225: 1410–1427 31442305 10.1111/nph.16134

[kiab060-B97] Pastori GM , del Río LA (1997) Natural senescence of pea leaves. Plant Physiol 113: 411–418 12223615 10.1104/pp.113.2.411 PMC158155

[kiab060-B98] Queval G , Issakidis-Bourguet E, Hoeberichts FA, Vandorpe M, Gakière B, Vanacker H, Miginiac-Maslow M, Van Breusegem F, Noctor G (2007) Conditional oxidative stress responses in the Arabidopsis photorespiratory mutant *cat2* demonstrate that redox state is a key modulator of daylength-dependent gene expression, and define photoperiod as a crucial factor in the regulation of H_2_O_2_-induced cell death. Plant J 52: 640–657 17877712 10.1111/j.1365-313X.2007.03263.x

[kiab060-B99] Queval G , Neukermans J, Vanderauwera S, Van Breusegem F, Noctor G (2012) Day length is a key regulator of transcriptomic responses to both CO_2_ and H_2_O_2_ in Arabidopsis. Plant Cell Environ 35: 374–387 21631535 10.1111/j.1365-3040.2011.02368.x

[kiab060-B100] Reumann S , Babujee L, Ma C, Wienkoop S, Siemsen T, Antonicelli GE, Rasche N, Lüder F, Weckwerth W, Jahn O (2007) Proteome analysis of Arabidopsis leaf peroxisomes reveals novel targeting peptides, metabolic pathways, and defense mechanisms. Plant Cell 19: 3170–3193 17951448 10.1105/tpc.107.050989 PMC2174697

[kiab060-B101] Reumann S , Bartel B (2016) Plant peroxisomes: recent discoveries in functional complexity, organelle homeostasis, and morphological dynamics. Curr Opin Plant Biol 34: 17–26 27500947 10.1016/j.pbi.2016.07.008 PMC5161562

[kiab060-B102] Reumann S , Bettermann M, Benz R, Heldt HW (1997) Evidence for the presence of a porin in the membrane of glyoxysomes of castor bean. Plant Physiol 115: 891–899 12223852 10.1104/pp.115.3.891 PMC158552

[kiab060-B103] Reumann S , Chowdhary G (2018) Prediction of peroxisomal matrix proteins in plants. Subcell Biochem 89: 125–138 30378021 10.1007/978-981-13-2233-4_5

[kiab060-B104] Reumann S , Ma C, Lemke S, Babujee L (2004) AraPerox. A database of putative Arabidopsis proteins. Plan Physiol 136: 2587–2608 10.1104/pp.104.043695 PMC523325 15333753

[kiab060-B105] Reumann S , Weber APM (2006) Plant peroxisomes respire in the light: Some gaps of the photorespiratory C_2_ cycle have become filled-Others remain. Biochim Biophys Acta 1763: 1496–1510 17046077 10.1016/j.bbamcr.2006.09.008

[kiab060-B106] Rinaldi MA , Patel AB, Park J, Lee K, Strader LC, Bartel B (2016) The roles of β-oxidation and cofactor homeostasis in peroxisome distribution and function in *Arabidopsis thaliana*. Genetics 204: 1089–1115 27605050 10.1534/genetics.116.193169 PMC5105844

[kiab060-B107] Rodríguez-Serrano M , Pazmiño DM, Sparkes I, Rochetti A, Hawes C, Romero-Puertas MC, Sandalio LM (2014) 2 ,4-Dichlorophenoxyacetic acid promotes *S-*nitrosylation and oxidation of actin affecting cytoskeleton and peroxisomal dynamics. J Exp Bot 65: 4783–4793 24913628 10.1093/jxb/eru237 PMC4144765

[kiab060-B108] Rodríguez-Serrano M , Romero-Puertas MC, Sanz-Fernández M, Hu J, Sandalio LM (2016) Peroxisomes extend peroxules in a fast response to stress via a reactive oxygen species-mediated induction of the peroxin PEX11a. Plant Physiol 171: 1665–1674 27208303 10.1104/pp.16.00648 PMC4936588

[kiab060-B109] Rodríguez-Serrano M , Romero-Puertas MC, Sparkes I, Hawes C, del Río LA, Sandalio LM (2009) Peroxisome dynamics in Arabidopsis plants under oxidative stress induced by cadmium. Free Radic Biol Med 48: 979 10.1016/j.freeradbiomed.2009.09.012 19765646

[kiab060-B110] Rojas CM , Senthil-kumar M, Wang K, Ryu C, Kaundal A, Mysore KS, Division PB, Roberts S, Foundation N (2012) Glycolate oxidase modulates reactive oxygen species-mediated signal transduction during nonhost resistance in *Nicotiana benthamiana* and Arabidopsis. Plan Cell 24: 336–352 10.1105/tpc.111.093245 PMC3289552 22286136

[kiab060-B111] Romero-Puertas MC , McCarthy I, Sandalio LM, Palma JM, Corpas FJ, Gómez M, del Río LA (1999) Cadmium toxicity and oxidative metabolism of pea leaf peroxisomes. Free Radic Res 31: 25–31 10.1080/10715769900301281 10694037

[kiab060-B112] Romero-Puertas MC , Rodríguez-Serrano M,, Corpas FJ, Gómez M, del Río LA, Sandalio LM (2004) Cadmium-induced subcellular accumulation of $O_{2}^{.-}$ and H_2_O_2_ in pea leaves. Plant Cell Environ 27: 1122–1134

[kiab060-B113] Romero-Puertas MC , Sandalio LM (2016) Nitric oxide level is self-regulating and also regulates its ROS partners. Front Plant Sci 7: 1–5 27014332 10.3389/fpls.2016.00316 PMC4795008

[kiab060-B114] Rosenwasser S , Fluhr R, Joshi JR, Leviatan N, Sela N, Hetzroni A, Friedman H (2013) ROSMETER: a bioinformatic tool for the identification of transcriptomic imprints related to reactive oxygen species type and origin provides new insights into stress responses. Plant Physiol 163: 1071–1083 23922270 10.1104/pp.113.218206 PMC3793026

[kiab060-B115] Rosenwasser S , Rot I, Sollner E, Meyer AJ, Smith Y, Leviatan N, Fluhr R, Friedman H (2011) Organelles contribute differentially to reactive oxygen species-related events during extended darkness. Plant Cell Environ 156: 185–201 10.1104/pp.110.169797 PMC3091045 21372201

[kiab060-B116] Ryan JM , Nebenführ A (2018) Update on myosin motors: molecular mechanisms and physiological functions. Plant Physiol 176: 119–127 29162634 10.1104/pp.17.01429 PMC5761821

[kiab060-B117] Sánchez-Vicente I , Fernández-Espinosa MG, Lorenzo O (2019) Nitric oxide molecular targets: reprogramming plant development upon stress. J Exp Bot 70: 4441–4460 31327004 10.1093/jxb/erz339 PMC6736187

[kiab060-B118] Sandalio LM , Gotor C, Romero LC, Romero-Puertas MC (2019) Multilevel regulation of peroxisomal proteome by post-translational modifications. Int J Mol Sci 20: 4881 10.3390/ijms20194881 PMC6801620 31581473

[kiab060-B119] Sandalio LM , Peláez-Vico MA, Romero-Puertas MC (2020) Peroxisomal metabolism and dynamics at the crossroads between stimulus perception and fast cell responses to the environment. Front Cell Dev Biol 8: 2014–2018 10.3389/fcell.2020.00505 PMC7333514 32676503

[kiab060-B120] Sandalio LM , Rodríguez-Serrano M, Romero-Puertas MC, del Río LA (2013) Role of peroxisomes as a source of reactive oxygen species (ROS) signaling molecules. Subcell Biochem 69: 231–225 23821152 10.1007/978-94-007-6889-5_13

[kiab060-B121] Sandalio LM , Romero-Puertas MC (2015) Peroxisomes sense and respond to environmental cues by regulating ROS and RNS signalling networks. Ann Bot 116: 475–485 26070643 10.1093/aob/mcv074 PMC4577995

[kiab060-B122] Schlicht M , Ludwig-m J, Burbach C, Volkmann D, Baluska F (2013) Indole-3-butyric acid induces lateral root formation via peroxisome-derived indole-3-acetic acid and nitric oxide. New Phytol 200: 473–482 23795714 10.1111/nph.12377

[kiab060-B123] Schmitz J , Rossoni AW, Maurino VG (2018) Dissecting the physiological function of plant glyoxalase I and glyoxalase I-like proteins. Front Plant Sci 9:1618 30483284 10.3389/fpls.2018.01618 PMC6240745

[kiab060-B124] Schrader M , Bonekamp NA, Islinger M (2012) Fission and proliferation of peroxisomes. Biochim Biophys Acta Mol Basis Dis 1822: 1343–1357 10.1016/j.bbadis.2011.12.014 22240198

[kiab060-B125] Schumann U , Prestele J, O’Geen H, Brueggeman R, Wanner G, Gietl C (2007) Requirement of the C3HC4 zinc RING finger of the Arabidopsis PEX10 for photorespiration and leaf peroxisome contact with chloroplasts. Proc Natl Acad Sci USA 104: 1069–1074 17215364 10.1073/pnas.0610402104 PMC1783365

[kiab060-B126] Sewelam N , Jaspert N, Van Der Kelen K, Tognetti VB, Schmitz J (2014) Spatial H_2_O_2_ signaling specificity: H_2_O_2_ from chloroplasts and peroxisomes modulates the plant transcriptome differentially. Mol Plant 7: 1191–1210 24908268 10.1093/mp/ssu070

[kiab060-B127] Shai N , Schuldiner M, Zalckvar E (2016) No peroxisome is an island-Peroxisome contact sites. Biochim Biophys Acta Mol Cell Res 1863: 1061–1069 10.1016/j.bbamcr.2015.09.016 PMC4869879 26384874

[kiab060-B128] Shibata M , Oikawa K, Yoshimoto K, Kondo M, Mano S, Yamada K, Hayashi M, Sakamoto W, Ohsumi Y, Nishimura M (2013) Highly oxidized peroxisomes are selectively degraded via autophagy in Arabidopsis. Plant Cell 25: 4967–4983 24368788 10.1105/tpc.113.116947 PMC3903999

[kiab060-B129] Sies H , Jones DP (2020) Reactive oxygen species (ROS) as pleiotropic physiological signalling agents. Nat Rev Mol Cell Biol 21: 363–383 32231263 10.1038/s41580-020-0230-3

[kiab060-B130] Sinclair AM , Trobacher CP, Mathur N, Greenwood JS, Mathur J (2009) Peroxule extension over ER-defined paths constitutes a rapid subcellular response to hydroxyl stress. Plant J 59: 231–242 19292761 10.1111/j.1365-313X.2009.03863.x

[kiab060-B131] Smirnoff N , Arnaud D (2019) Hydrogen peroxide metabolism and functions in plants. New Phytol 221: 1197–1214 30222198 10.1111/nph.15488

[kiab060-B132] Sousa RHV , Carvalho FEL, Lima-Melo Y, Alencar VTCB, Daloso DM, Margis-Pinheiro M, Komatsu S, Silveira JAG (2018) Impairment of peroxisomal APX and CAT activities increases protection of photosynthesis under oxidative stress. J Exp Bot 35: 627–639 10.1093/jxb/ery354 PMC6322566 30312463

[kiab060-B133] Su T , Li W, Wang P, Ma C (2019) Dynamics of peroxisome homeostasis and its role in stress response and signaling in plants. Front Plant Sci 10: 705 31214223 10.3389/fpls.2019.00705 PMC6557986

[kiab060-B134] Suzuki N , Koussevitzky S, Mittler RON, Miller GAD (2012) ROS and redox signalling in the response of plants to abiotic stress. Plan Cell Environ 35: 259–270 10.1111/j.1365-3040.2011.02336.x 21486305

[kiab060-B135] Talbi S Romero-Puertas MC Hernández A Terrón L Ferchichi A Sandalio LM (2015) Drought tolerance in a Saharian plant ***Oudneya* africa*na*: role of antioxidant defences. Environ Exp Bot 111: 114–126

[kiab060-B136] Terrón-Camero LC , Rodríguez-Serrano M, Sandalio LM, Romero-Puertas MC (2020) Nitric oxide is essential for cadmium-induced peroxule formation and peroxisome proliferation. Plant Cell Environ 43: 2492–2507 32692422 10.1111/pce.13855

[kiab060-B137] Thazar-Poulot N , Miquel M, Fobis-Loisy I, Gaude T (2015) Peroxisome extensions deliver the Arabidopsis SDP1 lipase to oil bodies. Proc Natl Acad Sci USA 112: 4158–4163 25775518 10.1073/pnas.1403322112 PMC4386359

[kiab060-B138] Tiew TW , Sheahan MB, Rose RJ (2015) Peroxisomes contribute to reactive oxygen species homeostasis and cell division induction in Arabidopsis protoplasts. Front Plant Sci 6: 1–16 26379686 10.3389/fpls.2015.00658 PMC4549554

[kiab060-B139] Tyutereva EV , Dobryakova KS, Schiermeyer A, Shishova MF, Pawlowski K, Demidchik V, Reumann S, Voitsekhovskaja OV (2018) The levels of peroxisomal catalase protein and activity modulate the onset of cell death in tobacco BY-2 cells via reactive oxygen species levels and autophagy. Funct Plant Biol 45: 247–258 32291039 10.1071/FP16418

[kiab060-B140] Umnajkitikorn K , Sade N, Rubio Wilhelmi MM, Gilbert ME, Blumwald E (2020) Silencing of OsCV (chloroplast vesiculation) maintained photorespiration and N assimilation in rice plants grown under elevated CO_2_. Plant Cell Environ 43: 920–933 31953871 10.1111/pce.13723

[kiab060-B141] Vandenabeele S , Kelen Van Der K, Dat J, Gadjev I, Boonefaes T, Morsa S, Van Breusegem F, Rottiers P, Slooten L, Van Montagu M, et al (2003) A comprehensive analysis of hydrogen peroxide-induced gene expression in tobacco. Proc Natl Acad Sci 100: 16113–16118 14671332 10.1073/pnas.2136610100 PMC307701

[kiab060-B142] Vandenabeele S , Vanderauwera S, Vuylsteke M, Rombauts S, Langebartels C, Seidlitz HK, Zabeau M, Van Montagu M, Inzé D, Van Breusegem F (2004) Catalase deficiency drastically affects gene expression induced by high light in *Arabidopsis thaliana*. Plant J 39: 45–58 15200641 10.1111/j.1365-313X.2004.02105.x

[kiab060-B143] Verslues PE , Batelli G, Grillo S, Agius F, Kim Y-S, Zhu J, Agarwal M, Katiyar-Agarwal S, Zhu J-K (2007) Interaction of SOS2 with nucleoside diphosphate kinase 2 and catalases reveals a point of connection between salt stress and H_2_O_2_ signaling in *Arabidopsis thaliana*. Mol Cell Biol 27: 7771–7780 17785451 10.1128/MCB.00429-07 PMC2169147

[kiab060-B144] Walker BJ , Vanloocke A, Bernacchi CJ, Ort DR (2016) The costs of photorespiration to food production now and in the future. Annu Rev Plant Biol 67: 107–129 26865340 10.1146/annurev-arplant-043015-111709

[kiab060-B145] Waller JC , Dhanoa PK, Schumann U, Mullen RT, Snedden WA (2010) Subcellular and tissue localization of NAD kinases from Arabidopsis: compartmentalization of de novo NADP biosynthesis. Planta 231: 305–317 19921251 10.1007/s00425-009-1047-7

[kiab060-B146] Walton PA , Brees C, Lismont C, Apanasets O, Fransen M (2017) The peroxisomal import receptor PEX5 functions as a stress sensor, retaining catalase in the cytosol in times of oxidative stress. Biochim Biophys Acta Mol Cell Res 1864: 1833–1843 28760655 10.1016/j.bbamcr.2017.07.013

[kiab060-B147] Wang W , Paschaladis K, Jian-Can F, Jie S, Ji-Hong L (2019) Polyamine catabolism in plants: a universal process with diverse functions. Front Plant Sci 10: 561 31134113 10.3389/fpls.2019.00561 PMC6513885

[kiab060-B148] Wang BL , Tang XY, Cheng LY, Zhang AZ, Zhang WH, Zhang FS, Liu JQ, Cao Y, Allan DL, Vance CP, et al (2010) Nitric oxide is involved in phosphorus deficiency-induced cluster-root development and citrate exudation in white lupin. New Phytol 187: 1112–1123 20553395 10.1111/j.1469-8137.2010.03323.x

[kiab060-B149] Werner AK , Witte C (2011) The biochemistry of nitrogen mobilization: purine ring catabolism. Trends Plant Sci 16: 381–387 21482173 10.1016/j.tplants.2011.03.012

[kiab060-B150] Wimalasekera R , Tebartz F, Scherer GFE (2011) Polyamines, polyamine oxidases and nitric oxide in development, abiotic and biotic stresses. Plant Sci 181: 593–603 21893256 10.1016/j.plantsci.2011.04.002

[kiab060-B151] Wu J , Shang Z, Wu J, Jiang X, Moschou PN, Sun W, Roubelakis-Angelakis KA, Zhang S (2010) Spermidine oxidase-derived H_2_O_2_ regulates pollen plasma membrane hyperpolarization-activated Ca^2+^ -permeable channels and pollen tube growth. Plant J 63: 1042–1053 20626657 10.1111/j.1365-313X.2010.04301.x

[kiab060-B152] Yang M , Li Z, Zhang K, Zhang X, Zhang Y, Wang X, Han C, Yu J, Xu K, Li D (2018) Barley stripe mosaic virus cb interacts with glycolate oxidase and inhibits peroxisomal ROS production to facilitate virus infection. Molecular Plant 11: 338–341 29066357 10.1016/j.molp.2017.10.007

[kiab060-B153] Yanik T , Donaldson RP (2005) A protective association between catalase and isocitrate lyase in peroxisomes. Arch Biochem Biophys 435: 243–252 15708367 10.1016/j.abb.2004.12.017

[kiab060-B154] Yoboue ED , Sitia R, Simmen T (2018) Redox crosstalk at endoplasmic reticulum (ER) membrane contact sites (MCS) uses toxic waste to deliver messages. Cell Death Dis 9: 331 29491367 10.1038/s41419-017-0033-4 PMC5832433

[kiab060-B155] Yoshimoto K , Shibata M, Kondo M, Oikawa K, Sato M, Toyooka K, Shirasu K, Nishimura M, Ohsumi Y (2014) Organ-specific quality control of plant peroxisomes is mediated by autophagy. J Cell Sci 127: 1161–1168 24463818 10.1242/jcs.139709

[kiab060-B156] Young PG , Bartel B (2016) Pexophagy and peroxisomal protein turnover in plants. Biochim Biophys Acta 1863: 999–1005 26348128 10.1016/j.bbamcr.2015.09.005 PMC4779433

[kiab060-B157] Young D , Pedre Ezeriņa D, De Smet B, Lewandowska A, Tossounian MA, Bodra N, Huang J, Astolfi Rosado L, Van Breusegem F, Messens J (2019) Protein promiscuity in H_2_O_2_ signaling. Antioxid Redox Signal 30: 1285–1324 29635930 10.1089/ars.2017.7013

[kiab060-B158] Yuan HM , Liu WC, Lu YT (2017) CATALASE2 coordinates SA-mediated repression of both auxin accumulation and JA biosynthesis in plant defenses. Cell Host Microbe 21: 143–155 28182949 10.1016/j.chom.2017.01.007

[kiab060-B159] Zhan N Wang C Chen L Yang H Feng J Gong X Ren B Wu R Mu J Li Y, . ( et al 2018) S-nitrosylation targets gsnoGSNO reductase for selective autophagy during hypoxia responses in plants. Mol Cell 71: 142–154 30008318 10.1016/j.molcel.2018.05.024

[kiab060-B160] Zhang T , Ma M, Chen T, Zhang L, Fan L, Zhang W, Wei B, Li S, Xuan W, Noctor G, et al (2020) Glutathione-dependent denitrosation of GSNOR1 promotes oxidative signalling downstream of H_2_O_2_. Plant Cell Environ 43: 1175–1191 31990075 10.1111/pce.13727

[kiab060-B161] Zhang Z , Xu Y, Xie Z, Li X, He Z, Peng X (2016) Association-dissociation of glycolate oxidase with catalase in rice: a potential switch to modulate intracellular H_2_O_2_ levels. Mol Plant 9: 737–748 26900141 10.1016/j.molp.2016.02.002

[kiab060-B162] Zhou J , Wang J, Cheng Y, Chi YJ, Fan B, Yu JQ, Chen Z (2013) NBR1-mediated selective autophagy targets insoluble ubiquitinated protein aggregates in plant stress responses. PLoS Genet 9: e1003196 23341779 10.1371/journal.pgen.1003196 PMC3547818

[kiab060-B163] Zou J , Li X, Ratnasekera D, Wang C, Liu W, Song L, Zhang W, Wu W (2015) Arabidopsis CALCIUM-DEPENDENT PROTEIN KINASE8 and CATALASE3 function in abscisic acid-mediated signaling and H_2_O_2_ homeostasis in stomatal guard cells under drought stress. Plant Cell 27: 1445–1460 25966761 10.1105/tpc.15.00144 PMC4456645

[kiab060-B164] Zhou YB , Liu C, Tang DY, Yan L, Wang D,, Yang YZ, Gui JS, Zhao XY, Li LG, Tang XD, et al (2018) The receptor-like cytoplasmic kinase STRK1 phosphorylates and activates CatC, thereby regulating H_2_O_2_ homeostasis and improving salt tolerance in rice. Plant Cell 30: 1100–1118 29581216 10.1105/tpc.17.01000 PMC6002193

